# Transcriptome Analysis of Low-Temperature-Treated Tetraploid Yellow *Actinidia chinensis* Planch. Tissue Culture Plantlets

**DOI:** 10.3390/life12101573

**Published:** 2022-10-10

**Authors:** Yipei Li, Zhiming Zhang, Xiaozhen Liu, Zhuo Wei, Xianang Zhang, Wen Bian, Shengxing Li, Hanyao Zhang

**Affiliations:** 1 Key Laboratory for Forest Resources Conservation and Utilization in the Southwest Mountains of China, Ministry of Education, Southwest Forestry University, Kunming 650224, China; 2 Key Laboratory of Biodiversity Conservation in Southwest China, National Forest and Glassland Administration, Southwest Forestry University, Kunming 650224, China

**Keywords:** yellow kiwifruit, tetraploid, cold tolerance, transcriptome, RT-qPCR analysis

## Abstract

**Simple Summary:**

Yellow kiwifruit (*Actinidia chinensis* Planch.) is popular in the market. However, it is highly susceptible to severe weather, including as low temperatures and frost, which may affect its production in the coming year. The cold-resistant mechanism of yellow kiwifruit associated with gene regulation is poorly investigated. To better understand cold-adaptive mechanisms, we grew plants under low-temperature conditions, which was followed by transcriptome analysis to discern the genes that play an active role in growth under low temperatures. The findings and dataset obtained in this study advance our knowledge of the cold-adaptive genes in regulatory networks and helps us to understand the cold-tolerance mechanisms in the tetraploid yellow kiwifruit.

**Abstract:**

The cold-resistant mechanism of yellow kiwifruit associated with gene regulation is poorly investigated. In this study, to provide insight into the causes of differences in low-temperature tolerance and to better understand cold-adaptive mechanisms, we treated yellow tetraploid kiwifruit ‘SWFU03’ tissue culture plantlets at low temperatures, used these plantlets for transcriptome analysis, and validated the expression levels of ten selected genes by real-time quantitative polymerase chain reaction (RT-qPCR) analysis. A number of 1630 differentially expressed genes (DEGs) were identified, of which 619 pathway genes were up-regulated, and 1011 were down-regulated in the cold treatment group. The DEGs enriched in the cold tolerance-related pathways mainly included the plant hormone signal transduction and the starch and sucrose metabolism pathway. RT-qPCR analysis confirmed the expression levels of eight up-regulated genes in these pathways in the cold-resistant mutants. In this study, cold tolerance-related pathways (the plant hormone signal transduction and starch and sucrose metabolism pathway) and genes, e.g., CEY00_Acc03316 (abscisic acid receptor PYL), CEY00_Acc13130 (bZIP transcription factor), CEY00_Acc33627 (TIFY protein), CEY00_Acc26744 (alpha-trehalose-phosphate synthase), CEY00_Acc28966 (beta-amylase), CEY00_Acc16756 (trehalose phosphatase), and CEY00_Acc08918 (beta-amylase 4) were found.

## 1. Introduction

Yellow kiwifruit (*Actinidia Chinensis* Planch.) has sweet and aromatic flesh as well as excellent nutrition and high economic value [1]. The fruit of yellow kiwifruit is popular in markets, and the output of yellow kiwifruit in New Zealand, Italy, Greece, Chile, and other countries has increased [1,2].

Cold injury is a problem affecting the growth of yellow kiwifruit [3]. The cold tolerance of yellow kiwifruit is not strong, so low temperatures and frost may result in yield reduction or plant death [4,5]. In addition to improving the cultivation techniques, breeding a cold-tolerant kiwifruit variety can fundamentally solve the problem of freezing injury. 

The low temperatures in winter may cause frostbite in kiwifruit, decreasing kiwifruit production [3,6]. Freezing injuries and late frost are disasters. The low-temperature freezing injury may cause some kiwifruit leaves to wilt and/or the flowers and fruits to fall, delay fruit production, reduce fruit quality, or result in the death of the whole plant [5,6]. To prevent freezing disasters, cultivation and selection from suitable environments and regions for planting, breeding, and cultivation of cold-resistant *A. chinensis* is a fundamental solution [3]. Therefore, it is urgently necessary to know how the kiwifruit plants react after treating with low temperatures.

RNA-Seq has the advantages of high accuracy, low restriction, high sequencing access, and high sensitivity, and it can uncover new genes, new transcripts, and the identification of mutations and shear sites [7,8,9]. Currently, transcriptome sequencing has been used extensively in studies related to cold resistance in plants [10,11,12]. The pathways involved in low-temperature response include plant hormone signal transduction, the calcium signaling pathway, the MAPK signaling pathway, starch, and sucrose metabolism [13,14]. The functional cold-resistance-related genes identified so far are genes of antioxidant systems such as SOD, genes related to phytohormone regulation, and genes of vital enzymes for fatty acid desaturation metabolism, and so on, but the cold resistance genes differ in different plants [11,12,15].

In this study, to provide insight into the causes of differences in low-temperature tolerance and to better understand cold-adaptive mechanisms, yellow tetraploid kiwifruit ‘SWFU03’ [16] tissue culture plantlets, which are known to be more tolerant to low temperatures [17], were treated with a low temperature and subjected to transcriptome analysis and validation of the expression levels of ten selected genes by real-time quantitative polymerase chain reaction (RT-qPCR) analysis. 

## 2. Materials and Methods

### 2.1. Materials

Yellow tetraploid kiwifruit ‘SWFU03’ plants were induced and identified by Li et al. [16], which were known to be more tolerant to low temperature. In vitro propagated tetraploid plants came from three mother tetraploid plants. The medium, grow chamber conditions, and photoperiod of the plantlets were described previously by Li et al. [16].

### 2.2. Transcriptome Analysis of Tetraploidy Yellow Kiwifruit

#### 2.2.1. Test Materials Preparing and RNA-Sequencing

Three bottles of tetraploid plantlets regenerated from explant about two months prior with 3 cm height were treated at 0 °C for 5 h (three plantlets in each bottle) as a treatment group, and three bottles of tetraploid plants were treated at 25 °C for 5 h (three plantlets in each bottle) as a control group. In the control group (C1, C2, and C3) and cold treatment group (T1, T2, and T3), three plantlets (one plant per bottle) were packed and labeled with tin foil. After sampling, the samples were quickly frozen in cryogenic liquid nitrogen and then placed in dry ice. Samples were sent to Anhui Microanalysis Gene Technology Co. Ltd. for transcriptome analysis. RNA-Seq was performed using Illumina HiSeqTM 2500 (San Diego, CA, USA).

#### 2.2.2. Sequence Data Filtering

After the libraries were constructed, the Illumina HiSeqTM 2500 platform was used for sequencing, and the sequence strategy was PE150. The original data (raw reads) obtained by sequencing were filtered, and the low-quality reads, connectors, and contamination, which accounted for more than 40% of the total read, were filtered out of the alkali base with a mass value of less than 15.

#### 2.2.3. Comparison of Reads Obtained by Sequencing with the Reference Genome

After the clean reads were obtained, they were compared with the whole genome sequence of *A. chinensis* [18] by HISAT2 version 2.1.0 [19], and the read mapping information was obtained. The usage rate of sequence data and the genetic relationship with the genome of *A. chinensis* were obtained by comparison.

#### 2.2.4. Detection of Differentially Expressed Genes (DEGs)

The screening threshold was FDR (false discovery rate) < 0.05, log2FC (fold change for a gene) > 1 or log2FC < −1. Based on the read count information, DEseq2 version 3.11 (or edgeR version 3.0.7) [20] software was used to analyze DEGs.

#### 2.2.5. Enrichment Analysis of DEGs in the GO Category and KEGG Pathway

The DEGs were annotated on the Gene Ontology database (GO, http://www.geneontology.org/ accessed on 11 March 2019) [20], and the GO term with FDR ≤ 0.05 was selected as the significantly enriched GO entry [20]. The DEGs were annotated on the Kyoto Encyclopedia of Genes and Genomes (KEGG) Pathway database [21].

#### 2.2.6. Screening of Differentially Expressed Genes

Differential gene data annotated to GO and KEGG were analyzed, and the cold tolerance-related genes in the kiwifruit were screened according to previous studies on cold tolerance genes and related pathways [21,22,23].

### 2.3. RT-qPCR Analysis

We selected three genes in the plant hormone signal transduction and seven in the starch and sucrose metabolism pathway, which were reported to be related to the low-temperature stress response [24,25,26], to check their transcriptional expression levels by RT-qPCR in low-temperature and room-temperature treated plantlets. The total RNA was extracted using the Qiagen RNeasy Mini Kit (Qiagen Inc., Valencia, CA, USA) and then reversely transcribed into cDNA by random primers. The RT-qPCR analysis was conducted according to a previous report [21]. Gene-specific primers were designed using Primer Premier 5.0 software (Premier Biosoft, Palo Alto, CA, USA), and the primers used for RT-qPCR analysis are listed in Table 1. The 2^(−^^ΔΔCt)^ method [21] was employed to analyze the data. We calculated the correlation coefficient and difference significances using Microsoft Excel 2007 (Microsoft Corporation, Redmond, WA, USA) [27].

## 3. Results

### 3.1. Effect of Cold Treatment on Tetraploid Yellow Kiwifruit

**Figure 1:**
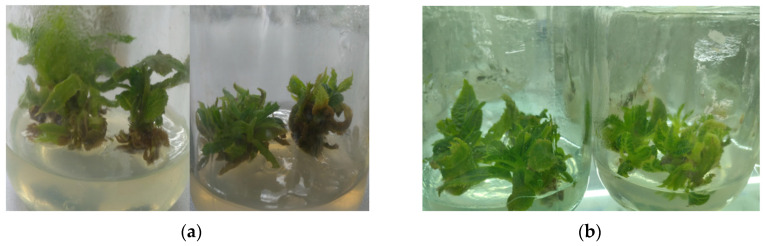
Changes in tetraploid plantlets under cold treatment. Note: (**a**) cold-treated plantlets; (**b**) control plantlets. The plantlets were kept at room temperature for 14 days after treatment at 0 °C for 12 h.

As shown in Figure 1, the freezing injury symptoms of the tetraploid plantlets were not very obvious compared to the control plants. The survival rate of tetraploid plantlets at 0 °C for 3 h was 88.89%. At 0 °C for 12 h, the survival rate of the tetraploid plantlets was 42.22%. After treatment with cold, the tetraploid plantlets were transferred to an MS medium and maintained at room temperature for observation. Fourteen days later, most of the tetraploid plantlets grew very well, and the degree of chilling injury was not very obvious. 

### 3.2. Results of Transcriptome Sequence Data Filtering

The original data of the samples are shown in Table 2. To improve the accuracy of the analysis results of the transcriptome data, the sequence data were filtered before the data analysis, and the filtered data are shown in Table 2. The sequence data of the sample transcriptome were of high quality.

### 3.3. Detection of DEGs

**Figure 2:**
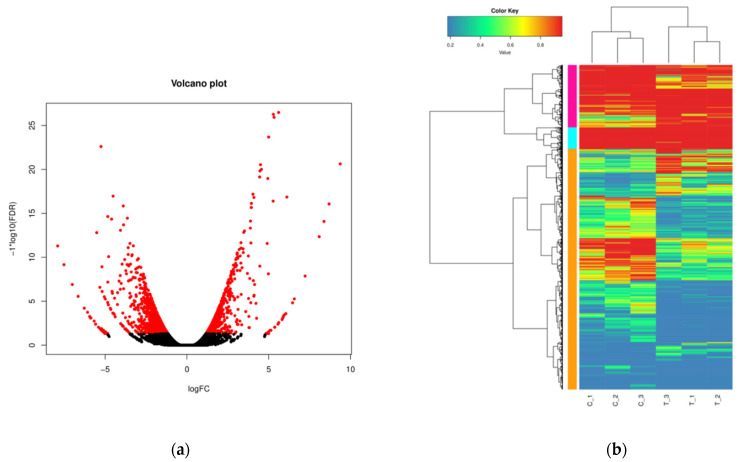
DEGs between cold-treated and control plantlets. Note: (**a**): red dot at the right side of 0, up-regulated DEGs in the cold treated plantlets compared to the control plantlets; red dot at the left side of 0, down-regulated DEGs. (**b**): C1, C2, and C3, control plantlets; T1, T2, and T3, cold-treated plantlets.

There were 1630 DEGs between the control group and the treatment group. By analyzing the DEGs between the control and treatment groups, we found that 619 DEGs were up-regulated and 1011 DEGs were down-regulated in the treatment group compared with the control group (Table 3, Figure 2).

### 3.4. Functional Classification and Enrichment Analysis of DEGs

**Figure 3:**
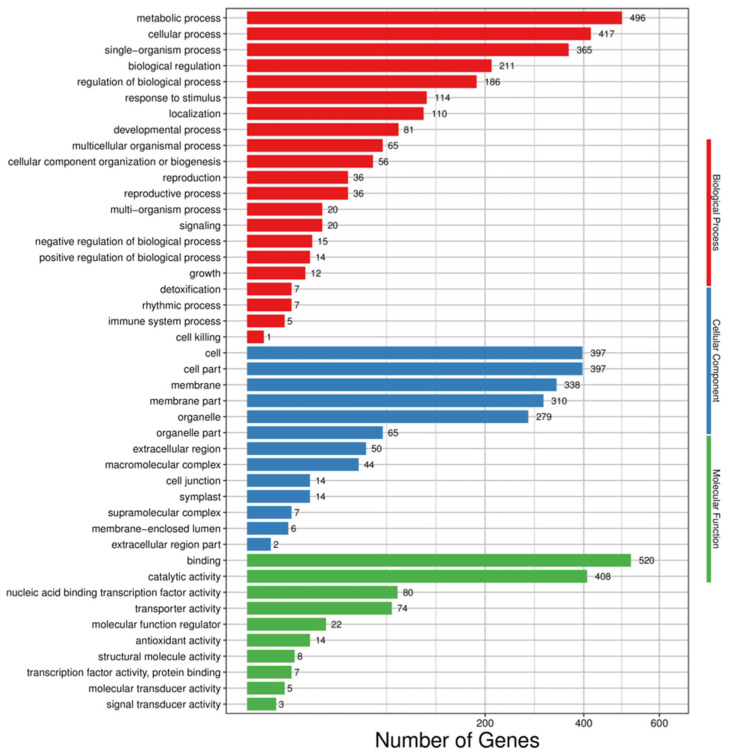
Bar graph of differentially expressed gene GO classification. Note that red bars represent the biological process, blue bars indicate the cellular component, and green bars represent the molecular function.

According to the results of differential gene detection, the DEGs were classified and enriched in the GO database. The results of GO enrichment analysis could be divided into three main functions, including molecular function, cellular component, and biological process. A total of 1025 DEGs were classified into 44 functional categories. There were ten functional classifications of molecular function, 13 classifications of cellular components, and 21 classifications of biological processes (Figure 3).

### 3.5. Functional Classification and Enrichment Analysis of the KEGG Pathway

Four hundred and ten differentially expressed genes were involved in 89 metabolic pathways in the control and treatment groups. The top five pathways enriching DEGs were the plant hormone signal transduction (including 25 DEGs), phenylpropanoid biosynthesis (24 DEGs), plant–pathogen interaction (20 DEGs), carbon metabolism (14 DEGs), and cysteine and methionine metabolism (13 DEGs). Among these, plant hormone signal transduction was the pathway with the largest number of DEGs annotated to this pathway, which belonged to environmental information processing in five branches.

The number of up-regulated DEGs enriched by phenylpropanoid biosynthesis was 16 in the first three pathways of the KEGG metabolic pathways and 14 DEGs in plant–pathogen interaction. A total of 11 DEGs were enriched by carbon metabolism. The top three down-regulation pathways were the plant hormone signal transduction, the enrichment of 19 DEGs, phenylpropanoid biosynthesis, enrichment of eight DEGs, and six DEGs enriched in carotenoid biosynthesis.

### 3.6. Screening and Analysis of Cold Tolerance-Related Genes

**Figure 4:**
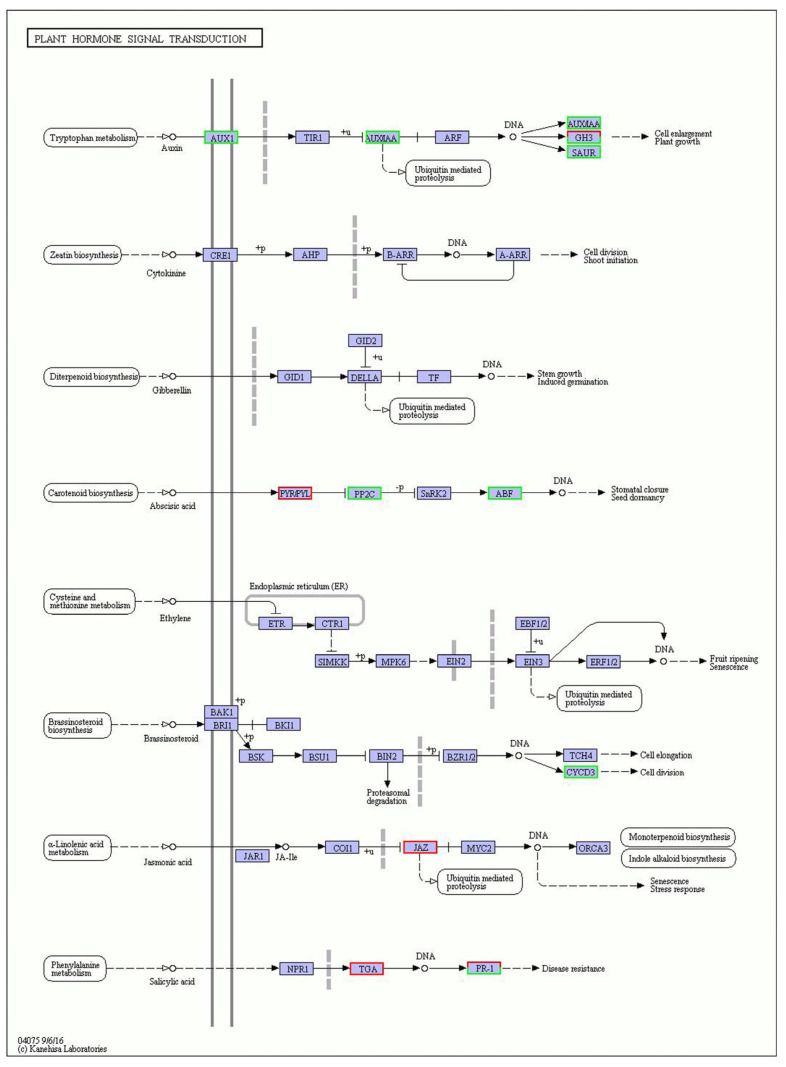
The plant hormone signal transduction pathway in *A. chinensis* tetraploid plants treated with cold. Note: red box, up-regulated genes; green box, down-regulated genes; blue box, both.

In this study, the largest number of DEGs was annotated to the plant hormone signal transduction pathway. Many cold tolerance-related studies have found that most DEGs are enriched in this pathway. The signal transduction pathway plays a vital role in the response of plants to low temperatures [28]. Therefore, the DEGs enriched in this pathway might be related to cold tolerance. The pathway map (KO04075) is shown in Figure 4.

There were 25 DEGs in the plant hormone signal transduction pathway, of which six were up-regulated. These were: CEY00_Acc07445 (disease-related leaf protein), CEY00_Acc03316 (abscisic acid receptor PYL), CEY00_Acc10294 (indole-3-acetic acid-amide synthetase), CEY00_Acc13130 (bZIP transcription factor family protein), CEY00_Acc33627 (TIFY protein), and CEY00_Acc21162 (pathogenesis-related protein); 19 DEGs were down-regulated, including CEY00_Acc07128 (auxin-induced protein), CEY00_Acc19743 (CICLE hypothetical protein), CEY00_Acc23775 (containing Cyclin_N domain protein), and CEY00_Acc07415 (indole-3-acetic acid-amide synthase). The genes involved in this pathway are summarized in Table 4.

Abscisic acid affects plant cold tolerance and gene expression regulation and can improve plant cold tolerance. Abscisic acid signal transduction was due to the interaction between abscisic acid receptors [29]. Abscisic acid receptor PYL was a receptor, and CEY00_Acc03316 was annotated as abscisic acid receptor PYL, so we inferred that CEY00_Acc03316 is a cold tolerance gene. The overexpression of bZIP transcription factor family proteins under stress was reported to improve the cold tolerance of plants [22,29]. Therefore, the CEY00_Acc13130 gene encodes a bZIP transcription factor family protein that may be a cold tolerance gene. The TIFY protein was induced and expressed at low temperature, and its gene family might be involved in low-temperature response and adaptation [30,31]. Therefore, we inferred that the CEY00_Acc33627 gene encoding a TIFY protein is a cold tolerance gene.

**Figure 5:**
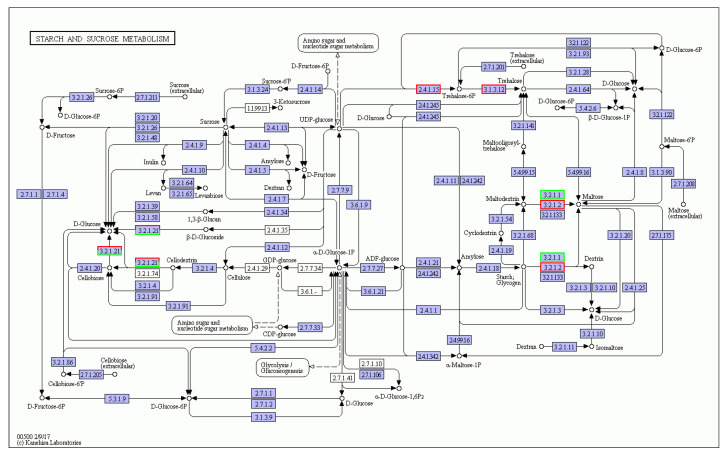
The starch and sucrose metabolic pathway in *A. chinensis* tetraploid plants treated with cold. Note: red box, up-regulated genes; green box, down-regulated genes; blue box, both.

The starch and sucrose metabolic pathways are related to cold tolerance [25,26]. Therefore, the DEGs of the starch and sucrose metabolism pathway were analyzed (Figure 5). There were eight DEGs in the starch and sucrose metabolism pathway, of which six were CEY00_Acc26744 (alpha-trehalose phosphate synthase), CEY00_Acc28966 (beta-amylase), CEY00_Acc16695 (beta-amylase), CEY00_Acc14271 (beta-glucosides GH1 family), CEY00_Acc16756 (trehalose phosphatase), and CEY00_Acc08918 (beta-amylase). There were two down-regulated DEGs, namely CEY00_Acc17108 (beta-glucosidase) and CEY00_Acc04508 (alpha-amylase) (Table 5). 

Trehalose-6-phosphate synthase can protect biological cell membranes and proteins from damage and is widely used in transgenic experiments with plants to improve their cold tolerance [22]. CEY00_Acc26744 and CEY00_Acc16756 were annotated as alpha-trehalose-phosphate synthase genes and inferred as cold-resistant genes. Maltose produced by starch decomposition catalyzed by beta-amylase may help protect the electron transport chain and protein in a stress environment. Cold tolerance of plants could be regulated by soluble sugar [32]. Beta-amylase might reduce cold stress injury by increasing the content of sugars and enhancing the cold tolerance of plants [33]. Hence, unigenes of CEY00_Acc28966 (beta-amylase), CEY00_Acc16695 (beta-amylase), and CEY00_Acc08918 (beta-amylase) were inferred as cold-resistant genes.

### 3.7. RT-qPCR Validation

**Figure 6:**
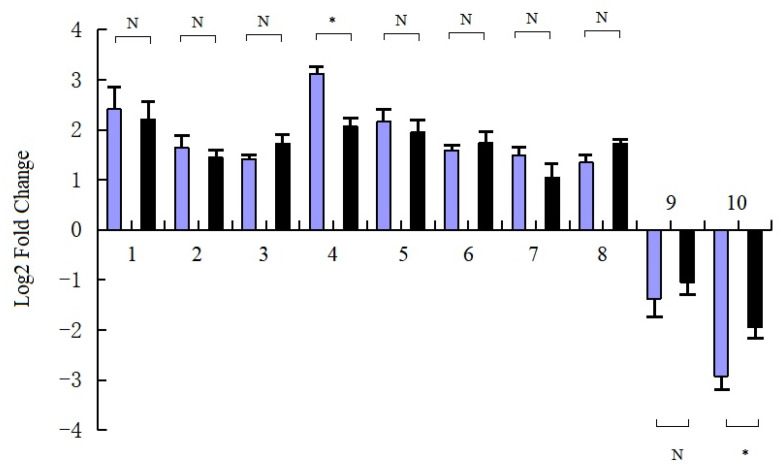
Comparison of RNA-sequencing and RT-qPCR results of selected DEGs. Note: 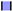, RNA-seq; 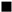, RT-qPCR. 1–10, genes encoding 1, abscisic acid receptor PYL; 2, bZIP transcription factor family protein; 3, protein TIFY; 4, alpha-trehalose-phosphate synthase; 5, beta-amylase; 6, beta-glucosidase 1 GH1 family; 7, trehalose-phosphate phosphatase F; 8, beta-amylase 4; 9, beta-glucosidase 12-like; 10, alpha-amylase. RT-qPCR was performed on 3 cold treated and 3 control plantlets, normalized with housekeeping gene elongation factor, and repeated 3 times. DEG, differentially expressed gene; RT-qPCR, real-time quantitative polymerase chain reaction. ∗, 0.01 *<* *p* *<* 0.05; N, >0.05.

The correlation coefficient (R^2^) between the expression levels of ten DEGs in RNA-seq and RT-qPCR is 0.6691, and the results of the RT-qPCR analysis of selected DEGs showed that the expression levels of genes were consistent with the transcriptome results (Figure 6). The difference in the expression level of two genes was significant, but that of eight genes was not significant. These ten selected genes were in the pathways related to the low-temperature stress response [24,25,26]. These findings indicated that the gene expression data obtained from the RNA-seq data of cold-treated and control plantlets were reliable. 

## 4. Discussion

The induction of polyploidy can improve the stress resistance of plants, such as cold resistance, drought resistance, and salt and alkali resistance [4]. Although the growth of polyploid plants is slow, stress tolerance and nutrient content are high [4,34,35]. Studies on polyploidy induction of fruit trees have been conducted for a long time, and polyploid plants, such as strawberries, kiwifruit, oranges, and pears, have contributed to the tolerance and the production of fruit trees [34,35]. However, not all plant polyploids are more resistant to abiotic stress than diploids. For example, Lu et al. [11] found that the freezing tolerance in *Solidago canadensis* decreased with increasing ploidy. In this study, most tetraploid plantlets grew very well, and the symptoms of chilling injury alleviated more than that of the diploid plantlets (data not shown). The cold resistance of tetraploid plantlets of yellow kiwifruit was higher than that of diploid plantlets.

Janská et al. [28] found that the plant signal transduction pathway plays a key role in the response of plants to low temperatures. In this study, we found that the DEGs enriched in this pathway were related to cold tolerance, which is consistent with the results by Janská et al. [28]. The starch metabolic pathway has been reported to be closely related to the cold tolerance response of plants [26]. In this study, the genes of the plant hormone signal transduction and starch and sucrose metabolism pathway were up in the treatment group, including CEY00_Acc03316 (abscisic acid receptor PYL), CEY00_Acc13130 (bZIP transcription factor), CEY00_Acc33627 (TIFY protein), CEY00_Acc26744 (alpha-trehalose-phosphate synthase), CEY00_Acc16756 (trehalose phosphatase), CEY00_Acc28966 (beta-amylase), CEY00_Acc16695 (beta-amylase), and CEY00_Acc08918 (beta-amylase). ABA has a certain effect on improving cold tolerance in plants [16]. Therefore, the differentially expressed gene CEY00_Acc03316 (abscisic acid receptor PYL) might play an important role in cold tolerance. 

The bZIP transcription factors play vital roles in regulating cold tolerance and other stresses [36]. Hence, CEY00_Acc13130 (bZIP transcription factor) might be a cold tolerance-related gene. Trehalose-6-phosphate synthase, a key enzyme in trehalose synthesis, was found to play a vital role in the cold response in different plant species [37,38]. Therefore, the alpha-trehalose-phosphate synthase (CEY00_Acc26744) in this study might be a cold-tolerance-related gene. Beta-amylase affects the cold tolerance of plants mainly by regulating the content of starch [39]. Beta-amylase-mediated starch degradation was found to play a vital role in the cold tolerance of plants [33]. In this study, DEGs encoding beta-amylase (CEY00_Acc28966, CEY00_Acc16695, and CEY00_Acc08918) might be cold tolerance genes in the kiwifruit.

Nowadays, the research on kiwifruit transcriptome analysis mainly focuses on the changes in fruit pulp color, fruit pigment, fruit development, and bacterial canker [40,41]. There are few studies on the stress tolerance of kiwifruit transcriptome analysis, particularly on cold tolerance, and there is no study on the cold-resistant genes of kiwifruit, which needs to be studied in the future. Genes related to cold tolerance identified in this study need further functional verification.

## 5. Conclusions

Based on the transcriptome analysis of tetraploid cold-resistant plants after cold treatment, two pathways related to cold tolerance were obtained, the plant hormone signal transduction and the starch and sucrose metabolism pathway. In these two pathways, seven DEGs related to cold tolerance, i.e., CEY00_Acc03316 (abscisic acid receptor PYL), CEY00_Acc13130 (bZIP transcription factor), CEY00_Acc33627 (TIFY protein), CEY00_Acc26744 (alpha-trehalose-phosphate synthase), CEY00_Acc28966 (beta-amylase), CEY00_Acc16756 (trehalose phosphatase), and CEY00_Acc08918 (beta-amylase 4) in kiwifruit were screened. These genes were confirmed to be up-regulated by RT-qPCR when the tetraploid plantlets were treated at low temperatures. 

## Figures and Tables

**Table 1:** Primer sequences of target genes (TG) and reference genes (RG) are used in RT-qPCR.

Gene Name	Primer	Sequence (5′–3′)	Length (bp)
Elongation factor (RG)	Forward primer	ACAAGCTGGTGACAATGTGG	127
	Reverse primer	CGACCACCTTCATCCTTTGT	
Abscisic acid receptor PYL(CEY00_Acc03316, TG)	Forward primer	GGTTTGGGAGGCTACTGAGT	149
	Reverse primer	TCCATTCGCATTCATCGCTG	
BZIP transcription factor (CEY00_Acc13130, TG)	Forward primer	TGTTTCTTGTGGATTGGCGG	332
	Reverse primer	TGCCCCATGTAGTTTCCCAT	
TIFY protein (CEY00_Acc33627, TG)	Forward primer	ATCCCCTGACCCTCCCTATT	237
	Reverse primer	CTCCGGGTTCATCTTCGAGA	
Alpha-trehalose-phosphate synthase (CEY00_Acc26744, TG)	Forward primer	TCGTCGGGGAATGATGATGT	225
	Reverse primer	GCATTCGATCAAACGGGTCA	
Beta-amylase (CEY00_Acc28966, TG)	Forward primer	ATGCTTGGTGGGGATTGGTA	226
	Reverse primer	GCCCGATCTGTCTGTGTAGA	
Beta-glucosidase 1 GH1 family (CEY00_Acc14271, TG)	Forward primer	GGGCCTCGGTGAAGTTTTAC	201
	Reverse primer	CCCCTTGATGTTGACTCCCT	
Trehalose phosphatase (CEY00_Acc16756, TG)	Forward primer	CGGTTGCGACTAACTCATGG	160
	Reverse primer	GCATCTTCGTCGGTCTTGTC	
Beta-amylase 4 (CEY00_Acc08918, TG)	Forward primer	CTTGGAGATGGCGAAGAAGC	149
	Reverse primer	TCTGTGTAGGCAAGGTCAGG	
Beta-glucosidase 12-like (CEY00_Acc17108, TG)	Forward primer	CCAAATTCACACCCGAGCAA	155
	Reverse primer	TTACCGAGGTGAGATTGGCA	
Alpha-amylase (CEY00_Acc04508, TG)	Forward primer	ACAGGATCAACACAGGCTCA	286
	Reverse primer	ATCGGCTGTTGAGGTCTTGA	

**Table 2:** Raw data and clean data output statistics.

Sample	Raw Reads num (Mb)	Raw Reads Base (Gb)	Raw Reads Q20 Rate (%)	Raw Reads Q30 Rate (%)	GC Rate (Mb)
C1	65.78	10.10	94.32	88.66	47.16
C2	66.96	9.80	94.8	89.28	47.04
C3	78.50	12.06	95.33	89.74	47.05
T1	64.12	9.84	94.00	88.06	47.27
T2	74.89	11.50	96.72	91.52	47.2
T3	81.16	12.47	95.67	90.2	47.44
**Sample**	**Clean Reads num (Mb)**	**Clean Reads** **Base (Gb)**	**Clean Reads** **Q20 Rate (%)**	**Clean Reads** **Q30 Rate (%)**	**rRNA Ratio (%)**
C1	59.86	9.13	96.85	91.58	0.06
C2	58.98	8.99	96.94	91.76	0.03
C3	74.14	11.29	96.91	91.57	0.03
T1	58.22	8.87	96.6	91.04	0.03
T2	73.76	11.25	97.17	92.07	0.03
T3	77.45	11.79	96.98	91.74	0.06

Note, C1, C2, and C3, control plantlets; T1, T2, and T3, cold treated plantlets.

**Table 3:** Statistical groups of differentially expressed genes in all comparison groups.

Group	Total	Up	Down
C-vs-T	1630	619	1011
C1-vs-T1	169	81	88
C2-vs-T2	281	133	148
C3-vs-T3	836	274	562

Note: Group, differential expression group; Total, the number of all DEGs; Up, the number of up-regulated DEGs; Down, the number of down-regulated DEGs. C, control group, and T, cold-treated group. C1, C2, and C3, control plantlets; T1, T2, and T3, cold-treated plantlets.

**Table 4:** Analysis of differentially expressed genes in plant hormone signal transduction and enrichment.

Gene ID	Gene Length	Gene Name	log2(FC)	Regulation
CEY00_Acc07445	648	Pathogenesis-related leaf protein	5.28215967	Up
CEY00_Acc03316	978	Abscisic acid receptor PYL	2.41039795	Up
CEY00_Acc10294	2193	Probable indole-3-acetic acid-amido synthetase GH3	1.78757476	Up
CEY00_Acc13130	1934	bZIP transcription factor family protein	1.63993265	Up
CEY00_Acc33627	1598	Protein TIFY	1.41856611	Up
CEY00_Acc21162	1191	Pathogenesis-related protein	1.38666594	Up
CEY00_Acc11766	1492	Cyclin D3-1	−1.36287325	Down
CEY00_Acc06865	671	The basic form of Pathogenesis-related protein	−1.38277358	Down
CEY00_Acc07128	1837	Amino acid transporter	−1.48608531	Down
CEY00_Acc19743	469	Hypothetical protein CICLE	−1.51643111	Down
CEY00_Acc06866	679	The basic form of pathogenesis-related protein	−1.52680253	Down
CEY00_Acc13744	2123	Indole-3-acetic acid-amido synthetase GH3.6	−1.67570429	Down
CEY00_Acc16267	524	Hypothetical protein CICLE	−1.72359172	Down
CEY00_Acc07415	2003	Indole-3-acetic acid-amido synthetase GH3.1	−1.74567531	Down
CEY00_Acc04564	706	Auxin early response protein SAUR	−1.75092162	Down
CEY00_Acc21097	1536	Cyclin-D3-1	−1.75509953	Down
CEY00_Acc10793	1871	Auxin transporter-like protein	−1.76951247	Down
CEY00_Acc10304	1525	Cyclin D3-2	−1.95276355	Down
CEY00_Acc32114	1035	Hypothetical protein VITISV	−2.16269734	Down
CEY00_Acc26372	2067	GH3 auxin-responsive promoter	−2.50991627	Down
CEY00_Acc04566	1001	Auxin-induced protein 6B-like	−2.75593648	Down
CEY00_Acc28473	851	Auxin-responsive protein IAA	−2.79532550	Down
CEY00_Acc23775	1717.72	Cyclin_N domain-containing protein	−2.82746855	Down
CEY00_Acc03517	1315	Hypothetical protein CICLE	−3.74278320	Down
CEY00_Acc07283	736	Auxin-induced protein 15A-like	−4.08073735	Down

**Table 5:** Analysis of DEGs in starch and sucrose metabolic pathways.

Gene ID	Gene Length (bp)	Gene Name	log2(FC)	Regulation
CEY00_Acc26744	2652	Alpha-trehalose-phosphate synthase	3.12212772	Up
CEY00_Acc28966	1926	Beta-amylase	2.16097859	Up
CEY00_Acc16695	1841	Beta-amylase	1.82925763	Up
CEY00_Acc14271	1766	Beta-glucosidase 1 GH1 family	1.59187712	Up
CEY00_Acc16756	1833	Trehalose-phosphate phosphatase F	1.49443763	Up
CEY00_Acc08918	1989	Beta-amylase 4	1.34221944	Up
CEY00_Acc17108	1465	Beta-glucosidase 12-like	−1.38324393	Down
CEY00_Acc04508	1435	Alpha-amylase	−2.92269962	Down

## Data Availability

All data generated or analyzed during this study are included in this published article. RNA-Seq data were presented at the short read archive (SRA) database of the National Center for Biotechnology Information (NCBI, accession number SAMN12612441).
